# Behavioural interventions targeting the prevention and treatment of young children’s mental health problems in low- and middle-income countries: a scoping review

**DOI:** 10.7189/jogh.15.04018

**Published:** 2025-01-24

**Authors:** Getachew Mullu Kassa, Zhiyuan Yu, Fentahun Minwuyelet, Deborah Gross

**Affiliations:** 1 Johns Hopkins University School of Nursing, Baltimore, Maryland, USA; 2 University of Pennsylvania School of Nursing, Philadelphia, Pennsylvania, USA; 3 Debre Markos University School of Nursing, Debre Markos, Ethiopia

## Abstract

**Background:**

Globally, 10% of children and adolescents live with mental health problems and often lack high-quality care. Over 80% of people facing mental health issues reside in low- and middle-income countries (LMICs). Failing to address children's mental health may prolong these challenges into adulthood, impeding their chances for a healthy life. This scoping review aims to describe the types, implementation strategies, effectiveness, and gaps of existing interventions for preventing and treating mental health problems in early childhood (<10 years) in LMICs.

**Methods:**

The study employed a scoping review of experimental studies published 2007−2023. Major databases including PubMed, Embase, Web of Science, and PsycINFO were searched using key terms related to the population (children), intervention (mental and/or behavioural health programmes), and outcome (mental health problems). Three authors independently conducted search strategy, article screening, data extraction, and quality assessment. The findings were presented using descriptive analysis and narrative synthesis.

**Results:**

Of 39 499 identified articles, 33 were included in the study, covering 7629 children and published between 2009−2022. Seventeen studies (51.5%) were from upper-middle-income countries, 13 (39.4%) were from lower-middle-income, and three (9.1%) were from low-income countries. Enrolment was community-based in 23 studies and health-facility based in 10 studies; the majority (79%) focused on children aged 3−8 years old. Almost two-third (63.6%) of studies were conducted in urban settings. Programmes encompassed various interventions such as parenting programmes (33.3%). A majority of studies (57.5%) employed group therapy for delivering the programme, with mental health professionals (21.2%) acting as the primary intervention providers. Interventions were primarily received by children (39.4%), followed by mothers/caregivers (33.3%). Most studies explored disruptive disorders (20 studies), social and behavioural problems (16 studies), and anxiety disorders (12 studies). Statistically significant intervention effects on child mental health outcomes were reported for 90% of published studies.

**Conclusions:**

Diverse behavioural programmes that improve the mental health of young children are available and effective in LMICs. Most interventions were delivered in urban settings and focus was on the use of health care professionals. Diverse intervention approaches, including parenting programmes and group therapy, were effective in addressing various mental health issues among young children.

The World Health Organization (WHO) defines mental health as a ‘state of well-being in which the individual realises his or her own abilities, can cope with the normal stresses of life, can work productively and fruitfully, and is able to make a contribution to his or her community’ [1]. Mental health is essential for a well-functioning society and fosters social capital and solidarity [2]. The status of the mental health of a population is the main determinant of the growth and development of a country [3]. Furthermore, the Lancet Commission on global mental health and sustainable development also recognises mental health as one of the fundamental human rights [4].

Childhood period is a critical stage of life for mental health, marked by rapid brain growth and development [5]. Childhood mental health problems are common mental health problems that can be diagnosed during childhood and include neurodevelopmental disorders such as attention-deficit/hyperactivity disorder (ADHD) and autism spectrum disorder, anxiety, and behaviour disorders [6].

Globally, one in eight people lives with a mental health problem, which is the leading cause of years lived with disability [7]. Despite the concerted international effort in the prevention and treatment of mental health problems, the global number of disability-adjusted life-years due to mental health problems increased from 80.8 million (3.1%) in 1990 to 125.3 million (4.9%) in 2019 [8]. According to a WHO report, mental health problems occur among 10% of children and adolescents globally; and most do not receive care [5]. More than 80% of people with mental health problems reside in low- and middle-income countries (LMICs) [9]. In addition, mental illness and substance use disorders are responsible for 8.8 and 16.6% of the total burden of disease in low-income and lower-middle-income countries, respectively [10]. However, 75% of individuals with mental, neurologic, and substance use disorders do not have access to the treatment they need [9].

According to a WHO report, mental health problems occur among 10% of children and adolescents globally and most do not receive care [5]. In sub-Saharan Africa (SSA) countries, one in seven children and adolescents have significant mental health problems, and one in 10 have a diagnosed psychiatric disorder [11]. The most common factors identified as contributing to children’s mental health problems in SSA were related to the family (e.g. maternal psychopathology, family disruption, and marital status), and childrearing context (e.g. poverty, trauma) [11]. Additional factors such as urbanisation, internal migration and lifestyle changes also increase the burden of mental illness in LMICs [12].

There is wide variability in the focus given to childhood mental health problems and mental health care in LMICs [12], with significant differences in the mental health workforce, financial resources, and infrastructure between high-income and low-income countries (LICs) [12,13]. Neglecting the mental health development of children increases the risk of extending mental health problems to adulthood and limits future opportunities for healthy and quality life [5]. Moreover, mental health problems also have enormous direct and indirect economic consequences. In 2010, the global economic cost attributed to mental health problems was 2.5 trillion USD and the cost is projected to increase to six trillion USD by 2030. From these, LMICs are projected to bear 35% of this total global cost [7].

World Health Organization published a Comprehensive Mental Health Action Plan 2013−2030 to promote mental health, prevent mental health problems, and achieve universal mental health service coverage [14]. One of the Sustainable Development Goals (SDG-3) aims to ensure healthy lives and promote well-being for all at all ages by 2030 [15]. Among the nine targets and four sub-targets of SDG-3 is a reduction of premature mortality from non-communicable diseases by one-third through prevention, treatment, and promotion of mental health and well-being [15]. This goal has been adopted by all United Nations members since 2015, and all countries are working towards achieving global and national health targets by 2030.

The health system and risk factors for mental health in LMIC are different from high-income countries [16]. This calls a need for specific prevention and treatment of mental health problems focusing on the LMIC population, particularly children. A previous review was conducted to assess mental health interventions among adolescents aged 10−19 years and in a limited context (SSA) [17]. The study excluded studies that were conducted among children birth to 10 years when most child mental health disorders emerge [18]. Similarly, another systematic review study was conducted to assess the effect of community mental health care in LMIC and used studies conducted among adults aged above 18 years and published between 1996−2006 [19].

Previous reviews have generally focused on broader mental health interventions, addressing a wide range of mental health issues across various populations [20–22]. In contrast, our scoping review specifically targets early childhood mental health interventions in LMICs. The current review included studies published after 2007 and was conducted to summarise the existing intervention programmes for the prevention and treatment of mental health problems among young children (≤10 years). Furthermore, findings will identify gaps in the existing literature and inform the priority research questions and policies that focus on childhood mental health problems in LMIC.

## METHODS

### Protocol design

The design and conduct of this scoping review was following Preferred Reporting Items for Systematic Reviews and Meta-analyses (PRISMA) guideline for scoping reviews [23].

### Eligibility criteria

The review used the Population, Intervention, Comparators, and Outcome (PICO) criteria to search and include published studies as described below:

• Population – young children (birth to 10 years)

• Intervention – behavioural intervention programmes for prevention or treatment of childhood mental health problems diagnosis or symptoms were included in this review. Behavioural interventions refer to structured strategies aimed at modifying problematic behaviours and promoting positive behaviours in children [24]. These interventions include parenting programmes, group therapies (such as group cognitive behavioural therapy), and other interventions (such as school based mental health interventions, animal assisted interventions, music therapy and so on). Parenting programmes are defined as ‘a set of activities or services aimed at improving how parents approach and execute their role as parents, specifically their parenting knowledge, attitudes, skills, behaviours, and practices’ [25].

• Comparator – absence of the intervention programme

• Outcome **–** childhood mental health problems

• Types of studies – the review included experimental studies, such as randomised controlled trials (RCTs) and quasi-experimental studies (QES), including pre-post studies

• Context – studies conducted in LMICs were included. The list of these countries was obtained from the World Bank website [26].

• Time frame – the review included studies published 2007–2023. As reported in the Lancet’s publication on the mental health of young people [27], there were limited evidence on the effectiveness of psychosocial and pharmacological treatment programmes for the mental health of young people before 2007. According to the report, only 6% of countries in Africa, 33% from the Eastern Mediterranean, and 63% of Southeast Asian countries had a child and adolescent mental health plan. Furthermore, a previous systematic review is also available on the effect of community mental health care in LMIC and included studies published from 1996–2006 [19].

• Language – studies that were published in the English language were included.

### Exclusion criteria

The current study excluded scoping reviews, systematic reviews and/or meta-analyses, study protocols, case studies, news, and editorials. Research on autism spectrum disorder was also excluded due to previously published review studies conducted on the topic [28–30].

### Outcome definitions

According to WHO, childhood mental health problems include childhood epilepsy, developmental disabilities, depression, anxiety, and behavioural disorders [31]. Behavioural disorders also include ADHD and conduct disorders [32]. Additionally, the study also included the common childhood disorders following the Diagnostic and Statistical Manual of Mental Disorders (DSM) classification including bipolar depression and anxiety [33]. Therefore, for the current study, mental health problems were grouped into five categories based on the reported outcomes in the included studies: 1) anxiety disorders, 2) ADHD, 3) disruptive disorders, 4) social and behavioural problems, and 5) other mental health problems. Anxiety disorders encompassed anxiety, separation anxiety, agoraphobia, social phobia, stress, and total difficulties score. Attention-deficit/hyperactivity disorder outcomes included ADHD symptoms, hyperactivity and impulsivity, and inattention disorder. Disruptive disorders included conduct problems, disruptive behaviour, externalising behaviour, oppositional defiant disorder (ODD), and aggression disorders. Social and behavioural outcomes covered child behaviour problems, emotional problems, emotional regulation, peer problems, peer victimisation, peer interaction, prosocial behaviour, positive child behaviour, socialisation, and social competence. Similarly, additional outcomes reported in studies, such as depression, internalising problems, mental health score, obsessive-compulsive disorder (OCD), and posttraumatic stress disorder (PTSD), were also included and categorised under the ‘other mental health problems’ outcome.

### Search strategy and sources

We conducted a search for published studies in major databases including PubMed, Embase, Web of Science, and PsycINFO. Additional studies were included in the study by screening the references of already included studies. The search utilised various keywords and Medical Subject Headings terms, combined using Boolean operators like ‘OR’ or ‘AND’. The search terms comprised a combination of PICO criteria. The search history of the PubMed database is presented in Table S1 in the **Online Supplementary Document**.

### Study selection

The study selection process involved two steps: 1) title and abstract screening and 2) full-text review. During the title and abstract review, all studies were screened and assigned as ‘yes’, ‘no’ or ‘maybe’. Studies with ‘yes’ and ‘maybe’ options were selected for full-text review. During the full-text review, we used the prior eligibility criteria to select studies. Studies that were classified as ‘no’ at the full-text review stage were given a reason for exclusion. The study selection process was done independently by three authors (GK, FM, and ZY). The Covidence system was used to manage the duplicate removal and study selection process [34].

### Data extraction

Data were extracted from included studies using a tool that was developed by the authors. The tool was piloted considering the research questions of the review. The tool included information on author name, publication year, study design, study area, data collection period, study setting, population, sample size, and other demographic characteristics of study participants. Additionally, data related to the type of treatment programme, intervention delivery modalities, implementation strategy, the effectiveness of the intervention programme in improving childhood mental health outcomes were extracted. The tool was developed in Qualtrics (Provo, Utah, USA, 2020), and data were extracted by three authors (GK, FM, and ZY) independently. Any discrepancies in the study selection and data extraction were resolved by consensus or by involving a third author. Moreover, for any missing data from the included articles, the authors of primary studies were contacted.

### Quality appraisal

The quality of included studies was assessed using the tools developed by the Joanna Brigs Institute (JBI) critical appraisal tool. For RCTs, the JBI appraisal criteria [35] contains 13 questions with four possible answer options: ‘Yes’, ‘No’, ‘Unclear’ or ‘Not Applicable.’ The focus of the questions includes the use of true randomisation for assignment to treatment groups, concealed allocation of treatment, similarity of treatment groups at baseline, blinding, completeness of the follow up period, similarity and validity of outcome measurement, and use of appropriate statistical method. Similarly, for quasi-experimental studies, the JBI’s critical appraisal checklist for quasi-experimental studies (non-randomised experimental studies) was utilised [36]. The QES tool contains nine questions, which focus on cause and effect, similarity of the population in the treatment and comparison, how the treatment programme is being delivered, the presence of a control group, multiple and similar ways of measurement of outcomes, completeness of the follow up, reliability, and use of appropriate statistical analysis method. Three authors (GK, FM, and ZY) independently gave responses to the quality appraisal of included studies.

### Data analysis and synthesis

The extracted data were collated and summarised using narrative synthesis and descriptive analysis methods. Tables and figures were used to present the findings of the study. The PRISMA flow diagram was produced using an online tool [37]. *R*-software (Vienna, Austria, 2024) was used for data-analysis including the analysis to show the trend of publications by year.

## RESULTS

### Selection of studies

**Figure 1:**
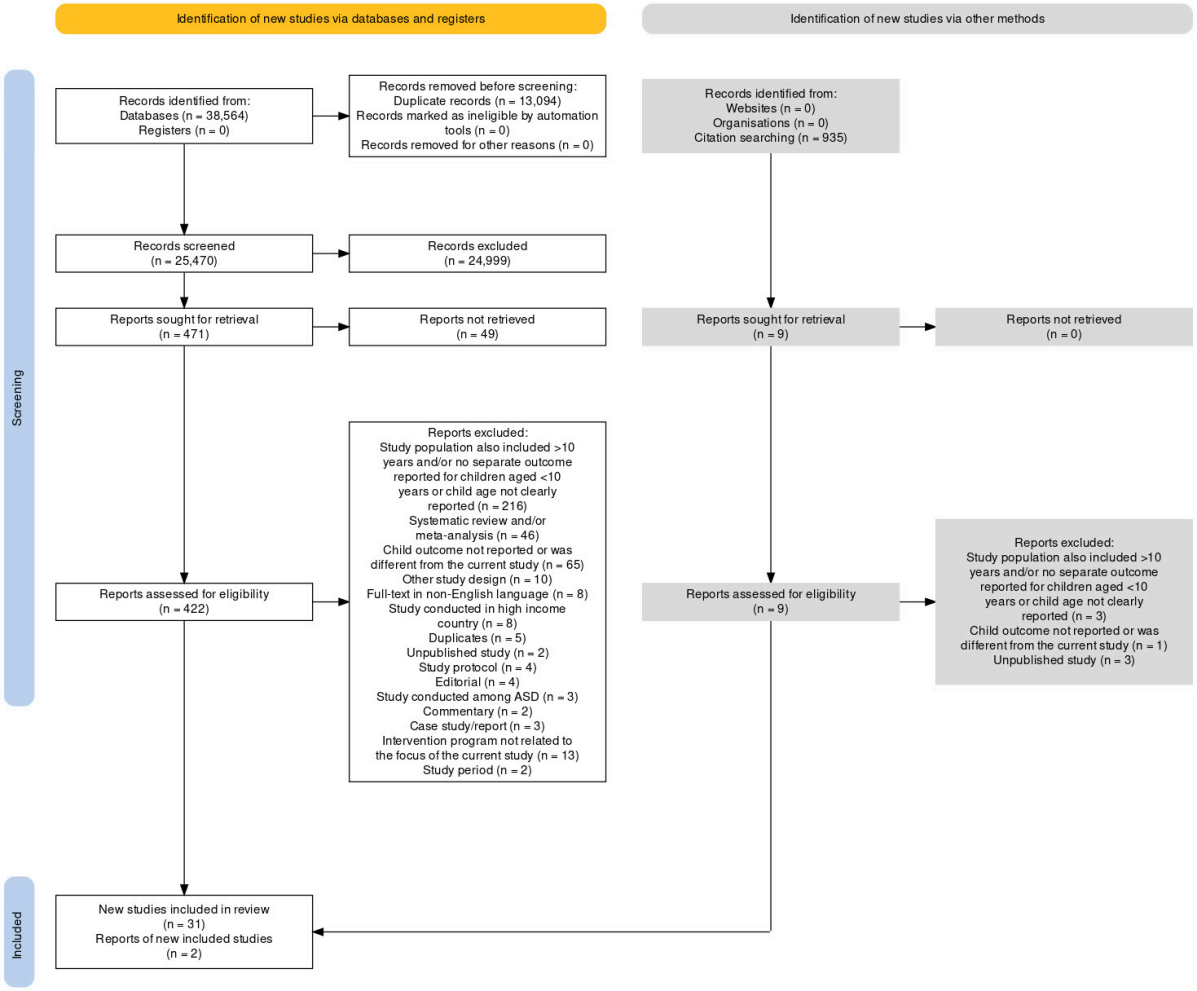
Flow diagram of inclusion process.

The search for studies resulted in a total of 39 499 studies. After removing ineligible studies due to duplication and reviewing titles and abstracts, we screened 431 full-text studies. Out of these, 398 studies were excluded. Finally, our study included 33 studies (**Figure 1**).

### Time of publication

**Figure 2:**
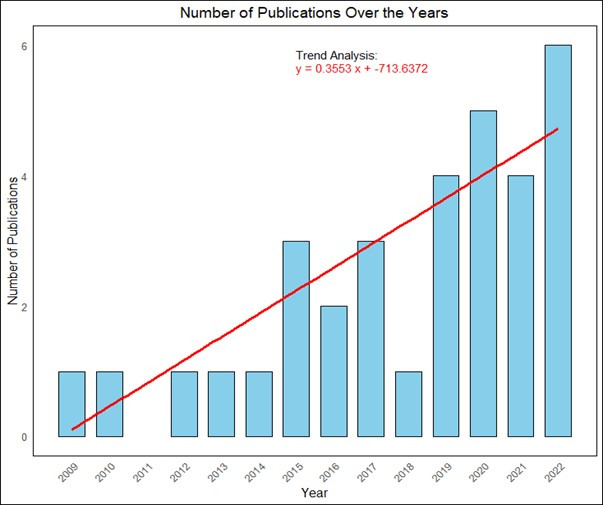
Distribution and trend of published studies over the years.

The included studies were published from 2009−2022, and the number of studies increased each year. As shown in **Figure 2**, 0−1 study was published annually from 2009−2014 and 4−6 studies were published annually from 2019−2022. Twenty-eight (84.5%) of included studies were published after 2015. The F-statistic for the linear trend analysis was 25.34 with a *P*-value <0.001.

### Study characteristics

Nine of the included studies were conducted in Iran [38–46], six in China [47–52], three in Brazil [53–55], three in South Africa [56–58], and two each in Pakistan [59,60], Turkey [61,62], and Jamaica [63,64]. The review also included studies from Bangladesh [65], India [66], Kenya [67], Liberia [68], Mexico [69], and Uganda [70]. Nine of the included studies were conducted in the Middle East and North Africa [38–46], six from East Asia and the Pacific [47–52], six from Latin America and the Caribbean [53–55,63,64,69], and six from SSA [56–58,67,68,70]. Additionally, four studies were from South Asia [59,60,65,66], and two were from Europe and Central Asia [61,62]. When disaggregated by the income category of countries, most studies (n = 17, 51.5%) were from upper-middle-income countries [47–58,61–64,69], 13 (39.4%) were from lower-middle-income countries [38,39,41–46,59,60,65–67], and three (9.1%) were from LICs [40,68,70]. Twenty-four of the included studies used a RCT design, of which 17 studies were randomised at individual level [41–46,48–52,54,56–58,64,68] and seven studies were randomised at the cluster level [38,47,53,59,60,63,70]. The remaining nine studies used quasi-experimental design, including pre-post design [39,40,55,61,62,65–67,69] (**Table 1**).

**Table 1:** Characteristics and setting of included studies

Author, year	Country	Income category	Study design	Study setting enrolment of participants	Study setting	Child age at enrolment into the study	Sex of included children
Bemanalizadeh et al., 2022	Iran	LMIE	IRCT	Outpatient public paediatrics clinic	Urban	up to 18 mo	Both male and female
Chu et al., 2022	China	UMIE	IRCT	School and hospital	Urban	6 − 8 y	Both male and female
Erdemir., 2022	Turkey	UMIE	QES	Summer school	Both Urban and Rural	5 − 6 y	Both male and female
Ndetei et al., 2022	Kenya	LMIE	Pre- and post-study	School	Rural and peri-urban	7 − 10 y	Both male and female
Tahan et al., 2022	Iran	LMIE	IRCT	Facility-based study	Urban	5 − 7 y	Not clearly reported
Zhu., 2022	China	UMIE	IRCT	Cooperative Hospital of Guangzhou University	Urban	2 − 7 y	Both male and female
Altafim et al., 2021	Brazil	UMIE	QES	Philanthropic educational centres, schools and community family health centres	Urban	3 − 8 y	Both male and female
Barik et al., 2021	India	LMIE	QES	Community-based study	Rural	3 − 8 y	Not clearly reported
Dowdall et al., 2021	South Africa	UMIE	IRCT	Church hall for the intervention group sessions and local research centre for all assessments	Urban	21 − 28 mo	Both male and female
Goudarzi et al., 2021	Iran	LMIE	QES	School	Urban	9 − 10 y	Only male
Daryabeigi et al., 2020	Iran	LMIE	IRCT	School	Urban	7 − 10 y	Only male
Rivero et al., 2020	Brazil	UMIE	IRCT	School	Urban	4 − 7 y	Both male and female
Zhang et al., 2020	China	UMIE	CRCT	Households	Rural	3 − 9 y	Both male and female
Maselko et al., 2020	Pakistan	LMIE	CRCT	Home	Rural	Up to 36 mo postnatal for children	Not reported
Ward et al., 2020	South Africa	UMIE	IRCT	Community-based study (home and school)	Peri-urban	2 − 9 y	Both male and female
Edrissi et al., 2019	Iran	LMIE	CRCT	Home/neighbour	Urban	4 − 6 y	Both male and female
Khademi et al., 2019	Iran	LMIE	IRCT	Psychiatry clinic	Urban	Preschool children with mean age and SD in the treatment and control group were 4.2 ± 1.09 and 3.9 ± 1.23 y, respectively	Both male and female
Morshed et al., 2019	Iran	LMIE	IRCT	Medical consultation centres	Urban	6 − 10 y	Both male and female
Pirnia et al., 2019	Iran	LMIE	IRCT	Community-based study	Rural	5 − 7 y	Both male and female
Akcan et al., 2018	Turkey	UMIE	QES	Kindergarten of a primary school	Not reported	Mean age of 63.4 mo for intervention and 63.8 mo for control group	Both male and female
Derakhshanpour et al., 2017	Iran	LIE	QES	Hospital/psychosocial support unit	Urban	Children <2 y to >6 y old	Both male and female
Goncalves et al., 2017	Brazil	UMIE	CRCT	School	Urban	6.5 − 8.1 y	Both male and female
Huang et al., 2017	Uganda	LIE	CRCT	School	Urban	4 − 8 y	Both male and female
Leung et al. 2016	China	UMIE	IRCT	Professional rehabilitation service centres for clients with special needs	Urban	2 − 5 y	Both male and female
Li et al., 2016	China	UMIE	IRCT	Kindergarten school	Urban	4 − 5 y	Both male and female
Deeba et al., 2015	Bangladesh	LMIE	QES	Shelter-homes	Semi-urban areas	5 − 9 y	Both male and female
Maselko et al., 2015	Pakistan	LMIE	CRCT	Mother's home, child’s school or in the local community health worker’s house	Rural	IQR of 7.5 − 7.7 y at final follow-up period. The baseline was at third trimester of the mother’s pregnancy	Both male and female
Puffer et al., 2015	Liberia	LIE	IRCT	Schools	Rural	3 − 7 y	Both male and female
Eloff et al., 2014	South Africa	UMIE	IRCT	Clinics for HIV positive women were attending	Urban	6 − 10 y	Both male and female
Leung et al., 2013	China	UMIE	IRCT	Service centres of early education, training centres, special child care centres, early education and child centres, and parent resource centres	Urban	Mean age and SD in the treatment and control group were 50.48 ± 12.31 and 49.74 ± 10.71 mo, respectively	Both male and female
Baker-Henningham et al., 2012	Jamaica	UMIE	CRCT	School	Urban	3 − 6 y	Both male and female
Walker et al., 2010	Jamaica	UMIE	IRCT	Main public maternity hospital	Urban	Infants with GA≥37 weeks	Both male and female
Ozer et al., 2009	Mexico	UMIE	QES	Households	Rural	2 − 5 y	Both male and female

CRCT – cluster randomised controlled trial, GA – gestational age, HIV – human immunodeficiency virus, IQR – interquartile range, IRCT – individual randomised clinical trials, LIE – low-income economy, LMIE – lower-middle-income economy, QES – quasi-experimental study, SD – standard deviation, UMIE – upper-middle-income economy

### Study settings and sample size

Most studies (n = 23, 69.7%) enrolled participants in the community including schools [39,46,50,53,54,61–63,67,68,70], households [38,47,56,59,69] or both [60]. Study participants were also enrolled from shelter homes [65], church halls and research centres [58], combination of schools and health facilities [51,55], and other community settings [43,66]. Ten studies enrolled study participants from health facilities, including hospitals [40,44,52,64], paediatric clinics [41], counselling centres [42,45], early education service centres [49], rehabilitation centres [48], and clinics for women with human immunodeficiency virus (HIV) [57].

The majority of studies were conducted in urban settings (n = 21, 63.6%) [38–42,44–46,48–55,57,58,63,64,70]. Seven (21.2%) were conducted in rural areas [43,47,59,60,66,68,69] and four (12.1%) in diverse settings, including semi-urban [65], both urban and rural [62], peri-urban [56], and combinations of rural and peri-urban areas [67]. The total sample size at baseline for all of the included studies was 7629, with individual study sample sizes ranging from 16 − 1072 participants (**Table 1**).

### Parent/caregiver population

The study populations reported in the studies mainly included mothers/parents/caregivers [38,40,43–50,53–56,58,61,63–65,68,70]. Five studies also included teachers as study participants [45,53,54,63,70]. The review also included studies involving pregnant women screened for moderate or severe symptoms of depression [59], third trimester pregnant women diagnosed with major depressive episode [60], parent or caregiver diagnosed with stage four metastatic cancer and cannabis dependence [43], and women attending HIV clinics [57].

### Child population

**Figure 3:**
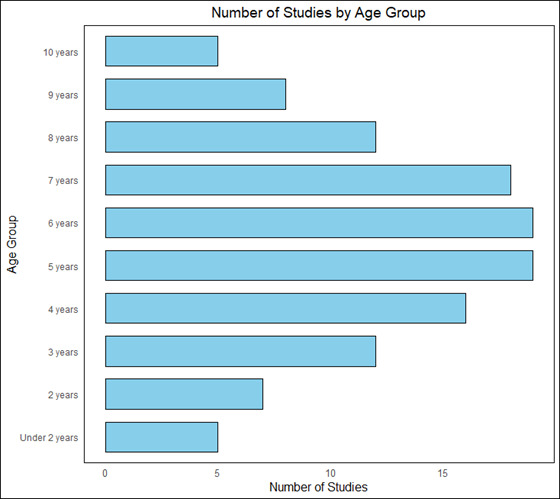
Number of studies by age of children at enrolment.

Twenty-eight (84.8%) studies included both male and female children, while two studies included only male children [39,46]. Child ages at enrolment ranged from 18 months [41] to 10 years [39]. The majority (n = 26, 79%) of the studies centred on children 3 − 8 years old with only 12 (36.4%) on children younger than three years old [40,41,48,49,52,56,58–61,64,69] and seven (21.2%) studies older than eight years old [39,45–47,56,57,65,67] (**Figure 3**).

Twelve studies did not report a specific mental health problem among children at enrolment [39,41,47,53,54,58,61,62,67–70]. Additionally, children with no reported disorders but whose mothers were diagnosed with moderate or severe symptoms of depression [59], diagnosed for major depressive episode using DSM-IV-TR criteria [58], and those whose mothers were attending HIV clinics [57] were included. One study was conducted among low-birthweight and term-born infants [64]. The remaining 17 studies were conducted among children with diagnosed or reported mental health problems. Among these, two studies included children with diagnosed ADHD based on the DSM-IV [44,52], and one study focused on children with ADHD and internalising and externalising behaviour problems based on the mother’s report [55]. Another study included children diagnosed with ADHD based on the parent’s report [51]. Two studies were conducted among children with anxiety based on mother’s report using the Spence Children Anxiety Scale for parents [42] and Preschool Anxiety Scale [38]. Additionally, one study included children with parent-rated shyness scores and who were also nominated by their classroom teacher as being shy [50].

Studies involving children with ODD symptoms [45], aggression problems [43], conduct problems [56], disruptive behaviour [66], externalising disorders based on the mother’s report [46], children with the highest levels of teacher-reported conduct problems [63] were included. Children who witnessed or experienced at least one severe DSM-IV defined traumatic event, and were vulnerable to symptoms of posttraumatic stress reactions [65], children who had been abused (physical, emotional, or sexual abuse) who were referred to the psychosocial support unit for services [40], and studies involving children with confirmed diagnoses of neurologic impairment [49] and development disabilities [48] were also included.

### Intervention programmes

Twenty-three (69.7%) of the included studies implemented intervention programmes in community-based settings, including schools, homes, and places of worship [38,39,46,47,49–51,53,54,56,58–70]. Schools were the most common venue for intervention delivery [38,39,50,51,53,54,61–63,67,70]. In contrast, six (18.2%) studies conducted interventions in health-facility-based settings, including hospitals, health centres, and clinics [41,42,44,45,48,52].

The review identified a range of intervention programmes aimed at addressing child mental health problems. Specifically, 11 studies utilised parenting programmes [38,41,43,44,48,49,51,55,56,66,68]. Five of the parenting programmes were implemented in upper middle-income country, five in LMIC, and two of the parenting programmes were implemented in LIC. Additional intervention programmes such as conditional cash transfer [69], animal-assisted therapy [42], and teacher training programme [63] were also used (Table S2 in the **Online Supplementary Document**). Eleven (33.3%) of the evidence-based interventions (e.g. Triple P, Parent-Child Interaction Therapy, and Tuning in to Kids programmes) were those developed and validated in high income countries [38,43,44,47–49,54,55,63,65,66]. Thirteen of the interventions included only the children [39,42,45–47,50,52–54,62,63,65,67], 11 focused solely on the parents/caregivers [38,40,41,44,48,49,55,58–60,68,69], and in seven included both children and parents/caregiver recipients [43,51,56,57,61,64,66]. Of the 18 studies that included parents as intervention recipients, 11 studies also included fathers in the study [45,47–49,51,56,62,66–69].

The reviewed intervention programmes were delivered by a variety of experts. Seven of the studies were delivered by mental health professionals [38,40,44,48,54,55,70] followed by teachers [46,53,62,63,67], community health workers [57,60,64], research staff members [39,51,55], facilitators who completed basic schooling [58], lay librarians [68], paraprofessional community members with high school level education [56], peer volunteers [59], trained health care providers [41], and female social workers [49]. For studies conducted in rural areas, intervention implementers included community health workers [60], peers [59], trained facilitator [47], and librarian facilitator [68].

The majority of studies (n = 21, 63.6%) were conducted in urban areas, intervention programme for nine studies conducted in urban areas were implemented at community level [40–42,44,45,48,49,52,57,64]. From the studies that reported the type of behavioural therapy provided to the study participants, 19 studies reported the use of group-based interventions programme [38,39,41,44,46–49,52–54,57–59,62,65,67,68,70] and five reported the use of individualised therapy [40,42,50,60,66]. Two studies used both group and individual therapy [45,51].

Fidelity in behavioural interventions is crucial because it ensures consistent and accurate implementation. Without fidelity, variations in delivery can lead to inconsistent results, compromising the reliability of reported outcomes and obscuring the true effectiveness of the intervention [71]. However, only 14 (42.4%) of the studies [38,46,47,49,53–56,58,59,62,63,65,70] included in the current review assessed intervention fidelity. Additionally, based on report from six studies [44,48,52,53,60,62], the absence of fathers in the programme, brief duration of the programme, scarcity of intervention providers, cultural or contextual factors, and heterogeneity of the study population were the main barriers of intervention programme delivery.

### Mental health outcomes targeted

Twenty studies had assessed outcomes at two time points, at baseline and immediately post-intervention [39,42–44,46,47,49,51–53,55,61–64,67–70,72]. More than two-third (69.7%) of included studies evaluated the short-term impact (less than one-year follow-up) of the intervention programme on child mental health outcomes. Additionally, 13 studies assessed outcomes at multiple time points post intervention, ranging from six months to two years [38,40,41,45,48,50,54,56–60,65].

The review encompassed a range of child-related mental health outcomes, with 26 studies measuring two or more mental health problems in children. Disruptive disorders were included in 20 (60.6%) studies [40,41,43–45,48,51,53,55,57,58,60–63,66–70], followed by 16 (48.5%) studies that included social and behavioural problems. Depression was addressed in five studies [41,44,46,57,65]. The least studied mental health outcomes included OCD [42,60] and PTSD [65].

### Reported effectiveness of the interventions

Over 90% (n = 30) of the studies demonstrated the statistically significant improvements in one or more mental health outcomes among young children. Three studies reported a non-significant impact of the intervention programmes on mental health outcomes [54,59,68]. The data are presented in Table S2 in the **Online Supplementary Document**.

#### Effectiveness of interventions on anxiety disorders

**Figure 4:**
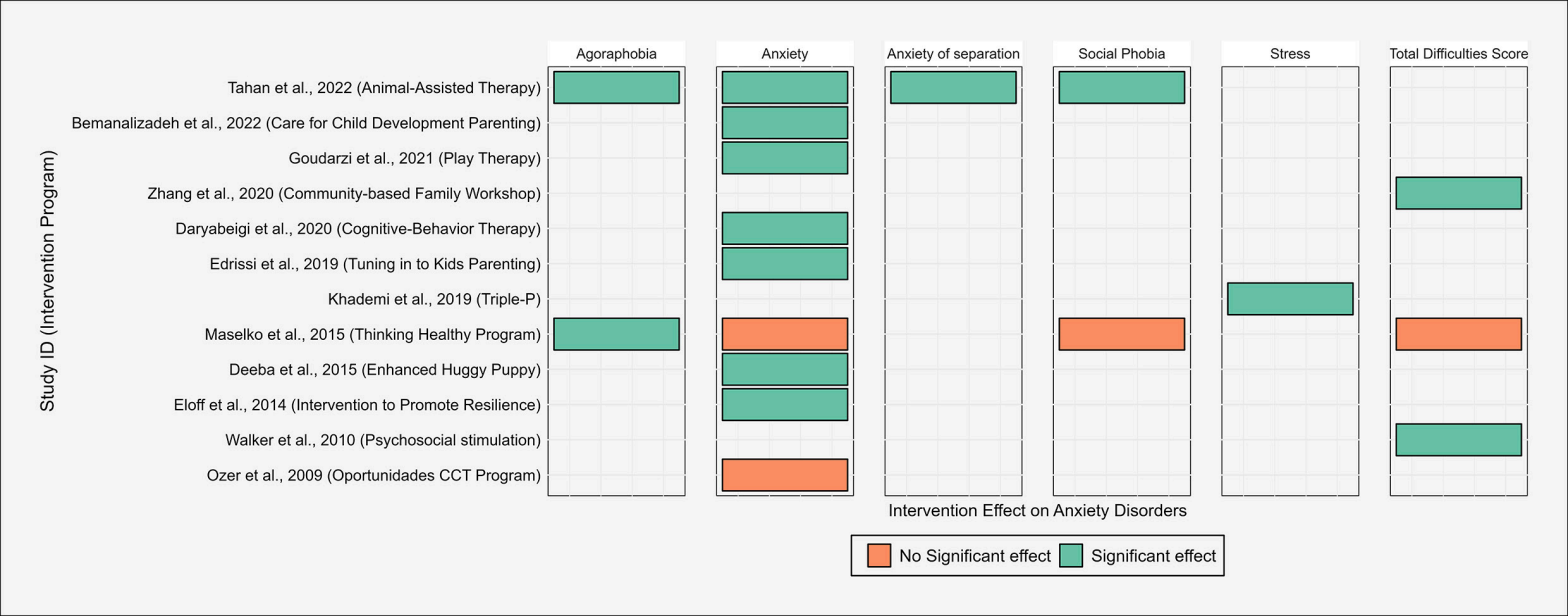
Distribution of studies and intervention programmes by effectiveness on anxiety disorders among young children in LMIC. LMIC – low- and middle-income countries.

A total of 12 studies evaluated the effectiveness of interventions on anxiety disorder-related outcomes [38,39,41,42,44,46,47,57,60,64,65,69]. Seven of the studies showed a significant impact of the intervention programmes on the anxiety level of children [38,39,41,42,46,57,65], and two studies were not significant [60,69]. From three studies that included total difficulties score as an outcome, two showed significant effect [47,64], and one showed non-significant effect [60] (**Figure 4**).

#### Effectiveness of interventions on ADHD

**Figure 5:**
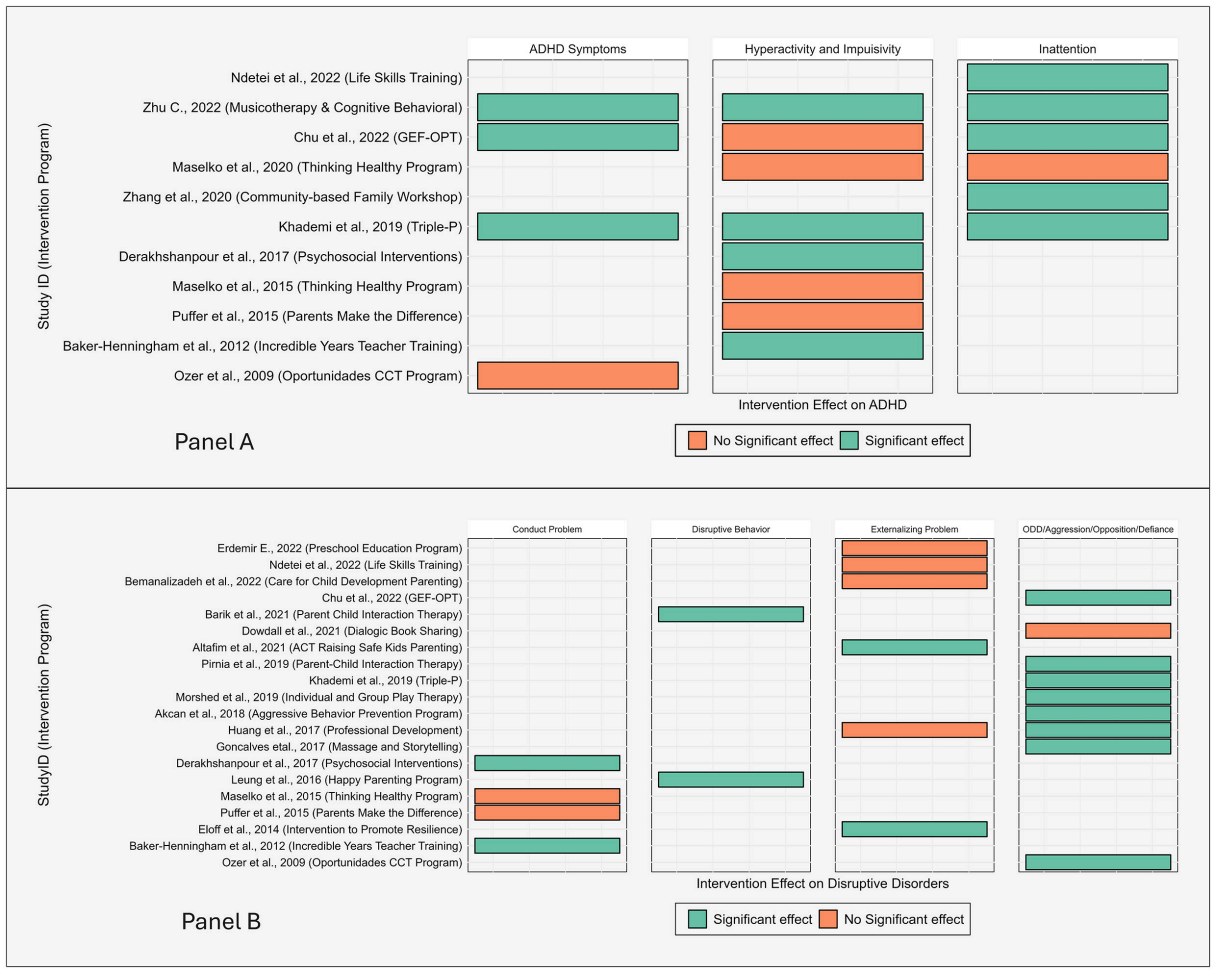
Distribution of studies and intervention programmes by effectiveness on ADHD and disruptive disorders among young children in LMIC. **Panel A**. Intervention effects on ADHD. **Panel B**. Intervention effects on disruptive disorders. ADHD – attention-deficit/hyperactivity disorder, LMIC – low- and middle-income countries.

A total of 11 studies evaluated the effect of behavioural intervention on ADHD [40,44,47,51,52,59,60,63,67–69]. From these, three out of the four studies that included ADHD symptoms [44,51,52], four out of the eight that included hyperactivity and impulsivity [40,44,52,63], and five out of the six that included inattention disorder [44,47,51,52,67] as outcomes showed a significant effect of the intervention programmes. Out of a total of 11 reported outcome assessments for ADHD-related outcomes in studies conducted in urban areas, interventions were effective in 10 outcomes reported in five studies [40,44,51,52,63] (**Figure 5****,** Panel A).

#### Effectiveness of interventions on disruptive behaviour disorders

From the 20 studies that evaluated the effect of behavioural intervention programmes on disruptive disorders [40,41,43–45,48,51,53,55,57,58,60–63,66–70], three studies showed a significant effect of the intervention on conduct problems [40,60,63], two on disruptive behaviour [48,66], two on externalising behaviour [55,57], and eight studies on ODD/aggression disorders [43–45,51,53,61,69,70] (**Figure 5****,** Panel B).

#### Effectiveness of interventions on social and behavioural problems

**Figure 6:**
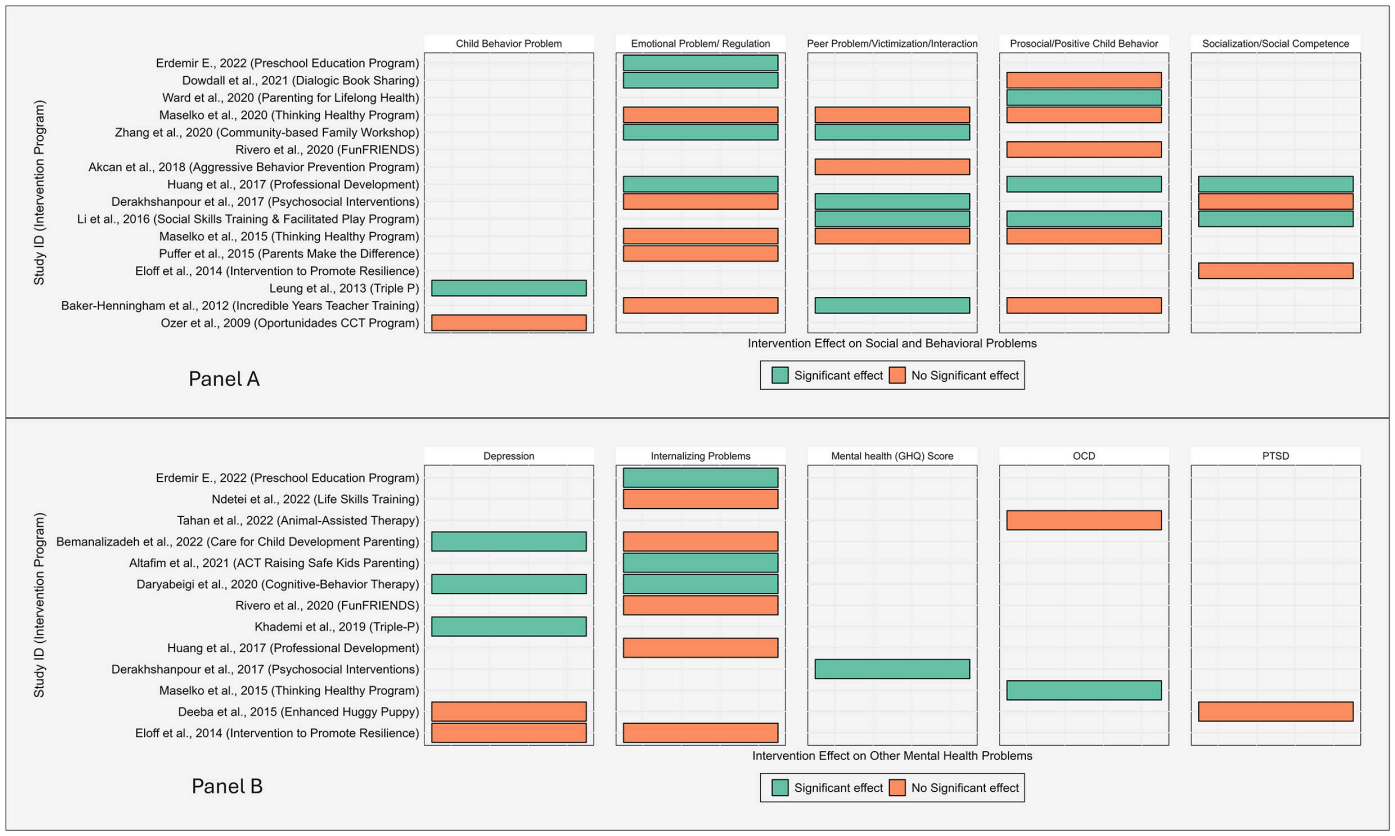
Distribution of studies and intervention programmes by effectiveness on social, behavioural, and other mental health problems among young children in LMIC. **Panel A**. Intervention effects on social and behavioural problems. **Panel B**. Intervention effects on other mental health problems. LMIC – low- and middle-income countries.

A total of 16 studies measured social and behavioural problems, encompassing five outcome categories. From these studies, one study [49] showed significant effect of the intervention programme on child behavioural problem, four on emotional problem/regulation outcome [47,58,62,70], four in peer problem/victimisation/interaction outcome [40,47,50,63], three in prosocial/positive child behaviour [50,56,70], and two in socialisation/social competence outcome [50,70] (**Figure 6****,** Panel A).

#### Effectiveness of interventions on other mental health problems

Five studies reported the effectiveness of behavioural intervention programmes on depression [41,44,46,57,65], eight studies on internalising problems [41,46,54,55,57,62,67,70], two studies on OCD [42,60], one study on mental health score using General Health Questionnaire [40], and one study on PTSD [65]. From these, three studies [41,44,46] out of the five showed significant effect of the intervention programme on depression outcome. Similarly, three out of the eight studies that included internalising problems as an outcome [46,55,62], one study on mental health [40], and one study on OCD [60] also showed a significant effect. However, the study [65] that included PTSD as an outcome showed a non-significant effect of the intervention programme (**Figure 6****,** Panel B).

### Critical appraisal of the studies

The critical appraisal scores for RCT studies ranged 7−10 out of a total of 13 criteria. Similarly, for QES, the scores ranged 6−9 out of a total of nine criteria (**Table 2**, **Table 3**).

**Table 2:** Critical appraisal of randomised controlled trial studies included in the scoping review on behavioural interventions for treatment and prevention of child mental health problems in low- and middle-income countries, 2007−2022

Author, year	C1*	C2*	C3*	C4*	C5*	C6*	C7*	C8*	C9*	C10*	C11*	C12*	C13*	Total
Bemanalizadeh et al., 2022	1†	1	1	0†	0	1	0	1	1	1	1	1	1	7
Chu et al., 2022	1	1	1	0	0	1	1	1	1	1	1	1	1	8
Tahan et al., 2022	1	0	1	0	0	1	0	1	1	1	1	1	1	7
Zhu, 2022	1	0	1	0	0	1	0	1	1	1	1	1	1	7
Dowdall et al., 2021	1	1	1	0	1	1	1	1	1	1	1	1	1	9
Daryabeigi et al., 2020	1	1	1	0	0	1	0	1	1	1	1	1	1	7
Rivero et al., 2020	1	1	1	0	0	1	0	1	1	1	1	1	1	7
Zhang et al., 2020	1	1	1	0	0	1	0	1	1	1	1	1	1	7
Maselko et al., 2020	1	0	1	0	0	1	1	1	1	1	1	1	1	8
Ward et al., 2020	1	1	1	1	1	1	1	1	1	1	1	1	1	10
Edrissi et al., 2019	1	0	1	0	0	1	0	1	1	1	1	1	1	7
Khademi et al., 2019	1	0	1	0	0	1	0	1	1	1	1	1	1	7
Morshed et al., 2019	1	0	1	0	0	1	0	1	1	1	1	1	1	7
Pirnia et al., 2019	1	0	1	1	1	1	1	1	1	1	1	1	1	10
Goncalves et al., 2017	0	1	1	0	0	1	1	1	1	1	1	1	1	8
Huang et al., 2017	1	0	1	1	0	1	1	1	1	1	1	1	1	9
Leung et al., 2016	1	0	1	0	0	1	0	1	1	1	1	1	1	7
Li et al., 2016	1	0	1	0	0	1	0	1	1	1	1	1	1	7
Maselko et al., 2015	1	0	1	0	0	1	1	1	1	1	1	1	1	8
Puffer et al., 2015	1	0	1	0	0	1	0	1	1	1	1	1	1	7
Eloff et al., 2014	1	0	1	0	0	1	0	1	1	1	1	1	1	7
Leung et al., 2013	1	0	1	0	0	1	0	1	1	1	1	1	1	7
Baker-Henningham et al., 2012	1	1	1	0	1	1	0	1	1	1	1	1	1	8
Walker et al., 2010	1	0	1	0	0	1	0	1	1	1	1	1	1	7

*C1 = Was true randomisation used for assignment of participants to treatment groups?; C2 = Was allocation to treatment groups concealed?; C3 = Were treatment groups similar at the baseline?; C4 = Were participants blind to treatment assignment?; C5 = Were those delivering the treatment blind to treatment assignment?; C6 = Were treatment groups treated identically other than the intervention of interest?; C7 = Were outcome assessors blind to treatment assignment?; C8 = Were outcomes measured in the same way for treatment groups?; C9 = Were outcomes measured in a reliable way?; C10 = Was follow-up complete and if not, were differences between groups in terms of their follow-up adequately described and analysed?; C11 = Were participants analysed in the groups to which they were randomised?; C12 = Was appropriate statistical analysis used?; C13 = Was the trial design appropriate and any deviations from the standard randomised controlled trials design (individual randomisation, parallel groups) accounted for in the conduct and analysis of the trial?

†1 = Yes, 0 = No.

**Table 3:** Critical appraisal of quasi-experimental studies included in the scoping review on behavioural interventions for treatment and prevention of child mental health problems in low- and middle-income countries, 2007–2022

Authors, year	C1*	C2*	C3*	C4*	C5*	C6*	C7*	C8*	C9*	Total
Erdemir E., 2022	1†	1	1	1	0†	1	1	1	1	8
Ndetei et al., 2022	1	1	1	0	1	1	1	1	1	8
Altafim et al., 2021	1	1	1	1	1	1	1	1	1	9
Barik et al., 2021	1	1	1	0	0	1	1	0	1	6
Goudarzi et al., 2021	1	1	1	1	0	1	1	1	1	8
Akcan et al., 2018	1	1	1	1	1	1	1	1	1	9
Derakhshanpour et al., 2017	1	1	1	0	0	1	1	1	1	7
Deeba et al., 2015	1	1	1	1	0	1	1	1	1	8
Ozer et al., 2009	1	1	1	1	1	1	1	1	1	9

*C1 = Is it clear in the study what is the cause and what is the effect (i.e. there is confusion about which variable comes first)?; C2 = Were the participants included in any comparisons similar?; C3 = Were the participants included in any comparisons receiving similar treatment/care, other than the exposure or intervention of interest?; C4 = Was there a control group?; C5 = Were there multiple measurements of the outcome both pre and post the intervention/exposure?; C6 = Was follow up complete and if not, were differences between groups in terms of their follow up adequately described and analysed?; C7 = Were the outcomes of participants included in any comparisons measured in the same way?; C8 = Were outcomes measured in a reliable way?; C9 = Was appropriate statistical analysis used?†1 = Yes, 0 = No

## DISCUSSION

The current scoping review was conducted to summarise existing intervention programmes for the prevention and treatment of early childhood mental health problems in LMICs. The review identified 33 studies with 7629 children, published 2009−2022. Half of the included studies were from upper-middle-income countries, and less than 10% of included studies were from low-income economy countries. The review encompassed a diverse range of community- and health-facility-based intervention programmes aimed at addressing child mental health problems, with one-third of the studies using a wide range of parenting programmes.

The increase in the number of publications from 2009−2022 reflects a growing recognition of the significance of prioritising child mental health programmes in LMICs. This trend may be linked to an increased awareness of mental health issues in children, evolving societal attitudes, and the changing landscape of health care and research in LMICs. Eighty-five percent of the studies included in the analysis were published after 2015, possibly due to the global push towards achieving the SDGs, particularly SDG-3, which aims to ensure healthy lives and promote well-being for all at all ages [73]. This global agenda has brought mental health issues, including those affecting children, to the forefront of health and development discussions [74]. Moreover, there is a growing recognition of the long-term impacts of mental health issues in children, with increasing evidence suggesting that if left unaddressed, these issues can lead to significant health, social, and economic challenges in adulthood [75].

The current review included a larger proportion (51.5%) of studies from upper-middle-income countries compared to 9% of studies from LICs. The variation in the number of studies across income categories could be attributed to the focus given to this area of research and a notable emphasis and disparity in research attention and investment in childhood mental health in these regions. This might be associated with the comparatively greater resources and infrastructure in upper-middle-income countries, resulting in a more robust research landscape in this domain. Additionally, the pattern could reflect disparities in health care infrastructure, funding availability, and awareness of childhood mental health issues. Therefore, the findings underscore the need for increased research focus and resource allocation in lower-income countries to address the existing imbalance and contribute to a more comprehensive understanding of intervention programmes aimed at improving childhood mental health.

According to the WHO’s 2020 report, only 51% of WHO member states reported that their mental health policies or plans aligned with international and regional human rights instruments. Additionally, just 52% of countries achieved the target related to mental health promotion and prevention programmes. When policies and plans incorporated assessments of necessary human and financial resources, only 39% of the surveyed nations confirmed the allocation of required human resources, and 34% reported the provision of the necessary financial resources [76]. Notably, the challenge is even more pronounced for countries with lower-income economy. These countries often face additional hurdles such as limited infrastructure, lack of trained professionals, and higher rates of poverty, which can exacerbate the difficulties in allocating necessary resources for early childhood mental health interventions [77]. Consequently, there is an urgent need for targeted interventions and increased global collaboration to address the specific challenges faced by lower-income countries in the realm of early childhood mental health intervention research.

Low- and middle-income countries often struggle with limited access to health care, particularly in the realm of mental health [78]. This challenge is attributed to various factors, including the unequal geographical distribution of the mental health workforce between urban and rural regions [79]. Additionally, the majority of individuals with mental, neurological, and substance use disorders in these countries do not receive adequate care. Research on mental health services in LMICs reveals that only 11.1% of individuals with severe mental health problems in China and 10.4% in Nigeria received evidence-based treatments in the past year [80]. This is compounded by inadequate awareness of mental health issues and the pervasive stigma surrounding them, factors that can deter individuals from seeking help [78]. Therefore, investing in preventive programmes is a crucial strategy to address these challenges and break the cycle of intergenerational mental health issues. Prevention and early intervention in mental health can mitigate the effects of mental illness, enabling individuals to live fulfilling, productive lives [81].

Most of the studies included in the review targeted mothers, with limited participation of fathers as study participants. This could be due to several factors, including the fact that mothers in many LMICs are the primary caregivers and spend more time with the child, making them more accessible for interventions [82]. Additionally, social and cultural norms in many LMICs, where gender inequalities are quite stark, often do not encourage or expect men to engage in nurturing caretaking roles [83]. Despite this, it is well-documented that fathers positively influence children’s early development [84] and maternal and child health outcomes [85] The underrepresentation of fathers in these programmes can negatively impact the outcomes of these interventions, as studies have shown that children’s outcomes improve when both parents are involved [86]. Therefore, addressing the research gap on the role of fathers and male caregivers and encouraging their central importance in their children’s well-being is essential [87]. Furthermore, strategies such as offering flexible schedules, creating father-friendly materials, and actively encouraging participation through targeted outreach could increase male caregiver involvement in behavioural interventions, such as parenting programmes [88].

The current study showed that one-third of the evidence-based interventions included in the current review were those developed and validated in high income countries. Interventions that are culturally adapted are more likely to be accepted and effective within diverse communities, and often result in higher implementation fidelity and social validity [89]. Without such adaptations, interventions may face resistance or be less effective, leading to disparities in outcomes. Therefore, incorporating cultural context into the development and delivery of these programmes is essential for achieving equitable and effective results. Furthermore, future studies need to explore how cultural and contextual factors could be integrated into mental health interventions in LMICs.

The findings of this study also underscore the importance of community- and health facility-based settings in the implementation of intervention programmes. In particular, 87 vs 100% of the studies that delivered the intervention programme at community and health facility settings, respectively, significantly improved at least one child mental health-related outcome. The variation could be attributed to the distinct objectives of the intervention programmes in both settings. Community-based interventions primarily focus on prevention, whereas health facility-level interventions are more treatment-oriented. Additionally, health facility-level interventions are typically administered by trained professionals in well-resourced environments, a contrast to the potentially limited resources in community settings [90,91].

Furthermore, a majority (70%) of the included studies implemented interventions in community settings such as schools, homes, and places of worship, with schools being the most common venue. This could be attributed to the accessibility and familiarity that these settings offer, potentially leading to higher participant engagement [92]. It also indicates the need to prioritise community-driven mental health initiatives, acknowledging their accessibility and potential for participant engagement. Moreover, such interventions can help combat stigma and raise public awareness about mental health issues, which are major barriers to seeking help in these settings [93,94]. On the other hand, a smaller proportion (18.2%) of studies opted for health-facility-based settings for their interventions. While health facility settings provide a more controlled environment, they may pose barriers such as distance and perceived stigma, potentially impacting participation rates and outcomes [95,96]. Furthermore, young children in LMICs face a range of challenges that can exacerbate mental health issues, including poverty, violence, and limited access to medical and psychological treatment [93,94]. Moreover, findings also call for investigating challenges in community- and health facility-based interventions for refining strategies and improving outcomes.

The review also highlighted a diverse range of intervention programmes aimed at addressing child mental health problems. Notably, one-third of the studies utilised parenting programmes, emphasising the influence parents, particularly mothers, have on the child’s mental health. There is a growing recognition of the importance of the family environment in shaping a child’s mental and behavioural health [97]. Parenting programmes can enhance the family environment by improving parenting skills, increasing parental mental health literacy, and reducing parental stress and mental health problems [97,98]. Additionally, parenting programmes are often more feasible and cost-effective to implement than individual child-focused interventions, especially in LMICs where resources for mental health are limited [98].

The current study also identified a diversity of interventionists involved in delivering behavioural intervention programmes for the prevention of mental health problems in LMICs. These experts include community health workers, trained facilitators, peer volunteers, teachers, trained mental health professionals, educational psychologists, and social workers. Community health workers have been found to be successful in improving the health of marginalised communities [99]. Moreover, stronger trainings for interventionists such as community health workers, peer volunteers, and teachers could improve the effectiveness of behavioural interventions [100]. Trained facilitators are also key to the successful implementation of behavioural intervention programmes as they help ensure that the intervention is delivered as intended and can adapt the programme to the local context [101]. Similarly, peer volunteers can play a significant role in behavioural intervention programmes by providing support, sharing experiences, and helping others navigate the system. Peer volunteers can also help reduce stigma and discrimination, which are major barriers to accessing mental health services [102]. Teachers and educational psychologists can also play a vital role in delivering behavioural interventions for children by identifying and supporting students who may be struggling with mental health issues [103,104]. Teachers are often the first to notice behavioural changes in students, making them key players in early intervention [105]. Additionally, social workers can provide a range of services to support individuals with mental health issues, including counselling, connecting individuals with resources, and advocating for their rights. However, the availability of educational psychologists and social workers and their implementation strategy can be challenging in LMIC health care systems [106].

The study also identified that a substantial proportion of the behavioural programmes reviewed were primarily targeted toward parents/caregivers, suggesting a recognition of the pivotal role played by parents and caregivers in influencing and shaping the mental health of children. This recognition potentially amplifies the impact of these interventions within the familial context. Some of the included studies also provided the intervention programme to children, with a deliberate effort to address behavioural challenges directly experienced by them. The involvement of both children and parents or caregivers in some of the behavioural interventions ensures a comprehensive approach to improving mental health. Evidence also suggests that comprehensive interventions targeting both children and parents/caregivers can lead to enduring positive outcomes [107]. Implementing such interventions can enhance early child cognitive, language, motor, and socioemotional development, strengthen attachment, and mitigate behaviour problems. Concurrently, it improves parenting knowledge, practices, and parent-child interactions [108]. However, findings of the current review showed that few of the studies evaluated whether positive effects were sustained beyond the immediate post-intervention period. Therefore, more studies evaluating the enduring effects of these interventions are also needed.

A large proportion of included studies targeted disruptive disorders, social and behavioural problems, anxiety, and ADHD. This emphasis can be attributed to their high prevalence and substantial impact on children’s development and overall well-being [109]. However, the attention given to evaluating the effectiveness of treatments for childhood outcomes such as OCD and PTSD was limited. Only one of the included studies [65] evaluated effectiveness of the intervention programme on PTSD outcome despite the high burden of PTSD in LMIC [110]. This limited focus to PTSD among children in LMICs could be attributed to limited mental health services [111] and overlapping of multiple adversities such as violence and food insecurity in LMICs [112]. Misdiagnosis of PTSD among children is also a common problem as it can be misclassified with other mental health problems such as ADHD and OCD [113]. Furthermore, the WHO report on world mental health survey also showed a 22.8 and 28.7% treatment seeking for PTSD among lower middle income and upper-middle income countries, respectively [114]. Therefore, there is a need for more research that evaluate effectiveness of behavioural interventions on PTSD.

Furthermore, LMICs are diverse in terms of culture, health care infrastructure, and economic status. These differences can significantly affect the implementation and success of mental health interventions for children. For instance, cultural variations can influence the acceptance and effectiveness of mental health programmes, while disparities in health care infrastructure can impact the availability and quality of services. Economic differences also play a crucial role, as they determine the resources available for mental health initiatives [115,116]. Recognising and addressing these diverse factors is essential for tailoring interventions to local contexts and improving their overall effectiveness [117,118].

Telehealth interventions for child mental and behavioural health have demonstrated substantial potential, particularly in improving access to care for families with young children who may encounter barriers to in-person services [119]. By overcoming health care infrastructure challenges in LMICs, telehealth can offer essential mental health support in areas where specialists are limited [120]. However, obstacles such as inadequate internet access, low digital literacy, and regulatory constraints can impede the successful implementation of telehealth in these settings [121]. Future research should aim to develop scalable, culturally appropriate telehealth models that address these challenges and enhance child mental health outcomes.

### Gaps in research

The study identified significant geographical disparities in the number of studies, with limited research conducted in low-income economy settings. Similarly, available studies primarily focus on urban settings, with limited research in rural areas. The intervention programmes mainly centre around the use of health care workers and in-person training programmes. Limited evidence was observed regarding the use of telehealth programmes to improve child mental health. Additionally, the intervention programme mainly focused on mothers, and there are limited studies that included fathers (n = 11, 33.3%) in the study. Even in these studies, the percentage of fathers who participated in the study was very low, <1% in some of the included studies [56]. Only 15% of studies included children aged under two years and children aged 10 years. More than two-third of included studies evaluated the immediate effect of the intervention programme on child mental health outcomes, and this may have limited our understanding of their long-term effectiveness. Furthermore, a majority of studies (57.6%) did not include fidelity assessment to evaluate adherence to the intervention programme.

### Strengths and limitations

The current review has certain strengths and limitations. One of the strengths lies in the comprehensive and broad scope across multiple databases employed in the search for relevant studies. The review also utilised standardised quality appraisal tools for evaluating the included studies. Furthermore, our review prioritised studies published after 2007, ensuring that the research synthesis of intervention programmes accurately reflects the current landscape of behavioural interventions for the prevention of childhood mental health problems in LMICs. However, the eligibility criteria of the studies may have inadvertently excluded relevant research, such as unpublished studies or those not indexed in the databases. The heterogeneity of the intervention programmes and the varied contexts in which they were implemented may make it challenging to draw definitive conclusions about their effectiveness.

Most of the studies included in this review were conducted in urban settings, highlighting a gap in the evaluation of behavioural interventions in rural and underserved areas. Future research should address this gap by prioritising studies in these regions to ensure more comprehensive and inclusive findings. Additionally, limitations such as potential biases within the included studies and variability in the quality and rigor of the interventions may have impacted the generalisability of the results. These factors should be considered when interpreting the findings, and future longitudinal and RCT studies are recommended in rural and underserved areas to enhance the robustness and applicability of outcomes across diverse populations. Furthermore, the focus on published studies in the current review may have excluded innovative or community-led interventions, digital programmes, and policy-level strategies that are not formally published or indexed in major databases. Future research should consider incorporating these less conventional sources to provide a more comprehensive view of the intervention landscape.

## CONCLUSIONS

This review has provided valuable insights into the landscape of intervention programmes targeting the mental health of children aged <10 years in LMICs. The increasing number of studies over the years highlights the growing recognition of the importance of addressing these critical issues, particularly in urban areas. Half of the included studies were conducted in upper-middle-income countries, with limited studies from LICs. The majority of included studies implemented intervention programmes in community-based settings, mainly at school. Additionally, almost one-third of studies utilised parenting programmes. Intervention programmes were delivered primarily by mental health professionals. The intervention programmes were mainly received by children, parents/caregivers, both parents/caregivers, and teachers. The majority of the studies showed the effectiveness of intervention programmes in improving the mental health outcomes of children. Furthermore, the absence of fathers in the programme, the brief duration of the programme, scarcity of intervention providers, and the heterogeneity of the study population were the main barriers to intervention programme delivery. Two-thirds of included studies measured two or more mental health outcomes in children, including disruptive disorders, social and behavioural problems, anxiety, and ADHD. Future studies focusing on rural areas and LICs, and the use of telehealth approaches are recommended. Future research should prioritise long-term evaluations to ensure that interventions provide lasting benefits and to strengthen the overall evidence on their effectiveness.

## Additional material


Online Supplementary Document
