# Supplementary material for: Behavioural interventions targeting the prevention and treatment of young children’s mental health problems in low- and middle-income countries: a scoping review
Source: J Glob Health. 2025 Jan 24;15:04018. doi: 10.7189/jogh.15.04018 (PMC11758180; doi:10.7189/jogh.15.04018)
Supplement: Online Supplementary Document [file jogh-15-04018-s001.pdf]

Table S1. PubMed article searches history on behavioral interventions for treatment and prevention of child mental health problems in low- and middle-income countries, 2007- 2023.

Search #	Query	Results	Date and time
1	Search: **(((((((Child[Title/Abstract])) OR (young people[Title/Abstract])) OR (children[Title/Abstract])) OR (childhood[Title/Abstract])) OR (early childhood[Title/Abstract])) OR (minor[Title/Abstract])** Filters: **from 2007 - 2023**	1,034,768	1/17/23 TIME: 14:30:23
2	Search: (((((((((((((((((((((Prevention[Title/Abstract]) OR (Promotion[Title/Abstract])) OR (Treatment[Title/Abstract])) OR (Program[Title/Abstract])) OR (Parenting program[Title/Abstract])) OR (Mental health service[Title/Abstract])) OR (School mental health service[Title/Abstract])) OR (health care[Title/Abstract])) OR (Health service[Title/Abstract])) OR (Community mental health service[Title/Abstract])) OR (Community mental health centers[Title/Abstract])) OR (Psychological[Title/Abstract])) OR (Social[Title/Abstract])) OR (Psychosocial care[Title/Abstract])) OR (Psychiatric procedures[Title/Abstract])) OR (community mental health services[MeSH Terms])) OR (mental health service[MeSH Terms])) OR (School mental health services[MeSH Terms])) OR (Community mental health centers[MeSH Terms])) OR (Psychiatric rehabilitation[MeSH Terms])) OR (Prevention[MeSH Terms])) OR (Treatment[MeSH Terms]) Filters: from 2007 - 2023	5,957.113	14:31:12
3	Search: ((((((((((((((((((((((((((((((((((((((Mental health[Title/Abstract]) OR (Mental disorder[Title/Abstract])) OR (Psychiatric disorder[Title/Abstract])) OR (Behavior disorder[Title/Abstract])) OR (Behavioral problem[Title/Abstract])) OR (Child Mental Disorder[Title/Abstract])) OR (Attention deficit hyperactivity disorder[Title/Abstract])) OR (Attention Deficit[Title/Abstract] AND Disruptive[Title/Abstract])) OR (Behavior Disorders[Title/Abstract])) OR (Disruptive disorder[Title/Abstract])) OR (ADHD[Title/Abstract])) OR (Conduct disorder[Title/Abstract])) OR (Depressive disorder[Title/Abstract])) OR (Anxiety[Title/Abstract])) OR (Depression[Title/Abstract])) OR (Depressive symptom[Title/Abstract])) OR (Depressive disorder[Title/Abstract])) OR (Mood disorder[Title/Abstract])) OR (Peer problem[Title/Abstract])) OR (Internalizing[Title/Abstract] AND externalizing[Title/Abstract])) OR (Psychiatric disorders[Title/Abstract])) OR (Developmental disability[Title/Abstract])) OR (Developmental disorder[Title/Abstract])) OR (Developmental Disabilities[Title/Abstract])) OR (neurodevelopmental disorders[Title/Abstract])) OR (intellectual disability[Title/Abstract])) OR (communication disorders[Title/Abstract])) OR (emotional disorder[Title/Abstract])) OR (Mental health[MeSH Terms])) OR (Mental disorders[MeSH Terms])) OR (child mental disorders[MeSH Terms])) OR (Depression[MeSH Terms])) OR (Anxiety[MeSH Terms])) OR (anxiety disorders[MeSH Terms])) OR (Attention Deficit Disorder with Hyperactivity[MeSH Terms])) OR (developmental disabilities[MeSH Terms])) OR (Neurodevelopmental Disorders[MeSH Terms])) OR (Attention Deficit and Disruptive Behavior Disorders[MeSH Terms]) AND (2007:2023[pdat])	1,104,338	14:42:21

4	Search: ((afghanistan[MeSH Terms]) OR (albania[MeSH Terms])) OR (algeria[MeSH Terms])) OR (american samoa[MeSH Terms])) OR (angola[MeSH Terms])) OR (argentina[MeSH Terms])) OR (armenia[MeSH Terms])) OR (azerbaijan[MeSH Terms])) OR (bangladesh[MeSH Terms])) OR (belarus[MeSH Terms])) OR (belize[MeSH Terms])) OR (benin[MeSH Terms])) OR (bhutan[MeSH Terms])) OR (bolivia[MeSH Terms])) OR (bosnia and herzegovina[MeSH Terms])) OR (botswana[MeSH Terms])) OR (brazil[MeSH Terms])) OR (bulgaria[MeSH Terms])) OR (burkina faso[MeSH Terms])) OR (burundi[MeSH Terms])) OR (CABO VERDE[MeSH Terms])) OR (cambodia[MeSH Terms])) OR (cameroon[MeSH Terms])) OR (CENTRAL AFRICAN REPUBLIC[MeSH Terms])) OR (chad[MeSH Terms])) OR (china[MeSH Terms])) OR (colombia[MeSH Terms])) OR (comoros[MeSH Terms])) OR (congo[MeSH Terms])) OR (CONGO, REP[MeSH Terms])) OR (CONGO, DEM. REP.[MeSH Terms])) OR (costa rica[MeSH Terms])) OR (cote d'ivoire[MeSH Terms])) OR (cuba[MeSH Terms])) OR (djibouti[MeSH Terms])) OR (dominica[MeSH Terms])) OR (dominican republic[MeSH Terms])) OR (ecuador[MeSH Terms])) OR (arab republic of egypt[MeSH Terms])) OR (el salvador[MeSH Terms])) OR (equatorial guinea[MeSH Terms])) OR (ERITREA[MeSH Terms])) OR (ESWATINI[MeSH Terms])) OR (ethiopia[MeSH Terms])) OR (fiji[MeSH Terms])) OR (gabon[MeSH Terms])) OR (gambia[MeSH Terms])) OR (georgia[MeSH Terms])) OR (ghana[MeSH Terms])) OR (grenada[MeSH Terms])) OR (guatemala[MeSH Terms])) OR (guinea[MeSH Terms])) OR (guinea bissau[MeSH Terms])) OR (guyana[MeSH Terms])) OR (haiti[MeSH Terms])) OR (honduras[MeSH Terms])) OR (india[MeSH Terms])) OR (indonesia[MeSH Terms])) OR (iran[MeSH Terms])) OR (iraq[MeSH Terms])) OR (jamaica[MeSH Terms])) OR (jordan[MeSH Terms])) OR (kazakhstan[MeSH Terms])) OR (kenya[MeSH Terms])) OR (kiribati[MeSH Terms])) OR (democratic people s republic of korea[MeSH Terms])) OR (kosovo[MeSH Terms])) OR (kyrgyz republic[MeSH Terms])) OR (laos[MeSH Terms])) OR (lebanon[MeSH Terms])) OR (lesotho[MeSH Terms])) OR (liberia[MeSH Terms])) OR (libya[MeSH Terms])) OR (madagascar[MeSH Terms])) OR (malawi[MeSH Terms])) OR (malaysia[MeSH Terms])) OR (maldives[MeSH Terms])) OR (mali[MeSH Terms])) OR (marshall islands[MeSH Terms])) OR (mauritania[MeSH Terms])) OR (mexico[MeSH Terms])) OR (micronesia, federated states of[MeSH Terms])) OR (moldova[MeSH Terms])) OR (montenegro[MeSH Terms])) OR (morocco[MeSH Terms])) OR (mozambique[MeSH Terms])) OR (myanmar[MeSH Terms])) OR (namibia[MeSH Terms])) OR (nepal[MeSH Terms])) OR (nicaragua[MeSH Terms])) OR (niger[MeSH Terms])) OR (nigeria[MeSH Terms])) OR (federal republic of nigeria[MeSH Terms])) OR (north macedonia[MeSH Terms])) OR (pakistan[MeSH Terms])) OR (papua new guinea[MeSH Terms])) OR (paraguay[MeSH Terms])) OR (peru[MeSH Terms])) OR (philippines[MeSH Terms])) OR (russian federation[MeSH Terms])) OR (rwanda[MeSH Terms])) OR (samoa[MeSH Terms])) OR (sao tome and principe[MeSH Terms])) OR (senegal[MeSH Terms])) OR	1,230,802	14:39:37

	(serbia[MeSH Terms])) OR (sierra leone[MeSH Terms])) OR (solomon islands[MeSH Terms])) OR (somalia[MeSH Terms])) OR (south africa[MeSH Terms])) OR (south sudan[MeSH Terms])) OR (sri lanka[MeSH Terms])) OR (st lucia[MeSH Terms])) OR (st vincent and the grenadines[MeSH Terms])) OR (sudan[MeSH Terms])) OR (suriname[MeSH Terms])) OR (syrian arab reublic[MeSH Terms])) OR (tajikistan[MeSH Terms])) OR (tanzania[MeSH Terms])) OR (thailand[MeSH Terms])) OR (democratic republic of timor leste[MeSH Terms])) OR (togo[MeSH Terms])) OR (tonga[MeSH Terms])) OR (tunisia[MeSH Terms])) OR (turkey[MeSH Terms])) OR (turkmenistan[MeSH Terms])) OR (tuvalu[MeSH Terms])) OR (uganda[MeSH Terms])) OR (republic of uganda[MeSH Terms])) OR (ukraine[MeSH Terms])) OR (uzbekistan[MeSH Terms])) OR (vanuatu[MeSH Terms])) OR (venezuela[MeSH Terms])) OR (vietnam[MeSH Terms])) OR (west bank[MeSH Terms])) OR (yemen[MeSH Terms])) OR (zambia[MeSH Terms])) OR (republic of zambia[MeSH Terms])) OR (zimbabwe[MeSH Terms])) OR (republic of zimbabwe[MeSH Terms])) OR (afghanistan[Title/Abstract])) OR (albania[Title/Abstract])) OR (algeria[Title/Abstract])) OR (american samoa[Title/Abstract])) OR (angola[Title/Abstract])) OR (argentina[Title/Abstract])) OR (armenia[Title/Abstract])) OR (azerbaijan[Title/Abstract])) OR (bangladesh[Title/Abstract])) OR (belarus[Title/Abstract])) OR (belize[Title/Abstract])) OR (benin[Title/Abstract])) OR (bhutan[Title/Abstract])) OR (bolivia[Title/Abstract])) OR (bosnia and herzegovina[Title/Abstract])) OR (botswana[Title/Abstract])) OR (brazil[Title/Abstract])) OR (bulgaria[Title/Abstract])) OR (burkina faso[Title/Abstract])) OR (burundi[Title/Abstract])) OR (CABO VERDE[Title/Abstract])) OR (cambodia[Title/Abstract])) OR (cameroon[Title/Abstract])) OR (CENTRAL AFRICAN REPUBLIC[Title/Abstract])) OR (chad[Title/Abstract])) OR (china[Title/Abstract])) OR (colombia[Title/Abstract])) OR (comoros[Title/Abstract])) OR (congo[Title/Abstract])) OR (CONGO, REP[Title/Abstract])) OR (CONGO, DEM. REP.[Title/Abstract])) OR (costa rica[Title/Abstract])) OR (cote d'ivoire[Title/Abstract])) OR (cuba[Title/Abstract])) OR (djibouti[Title/Abstract])) OR (dominica[Title/Abstract])) OR (dominican republic[Title/Abstract])) OR (ecuador[Title/Abstract])) OR (arab republic of egypt[Title/Abstract])) OR (el salvador[Title/Abstract])) OR (equatorial guinea[Title/Abstract])) OR (ERITREA[Title/Abstract])) OR (ESWATINI[Title/Abstract])) OR (ethiopia[Title/Abstract])) OR (fiji[Title/Abstract])) OR (gabon[Title/Abstract])) OR (gambia[Title/Abstract])) OR (georgia[Title/Abstract])) OR (ghana[Title/Abstract])) OR (grenada[Title/Abstract])) OR (guatemala[Title/Abstract])) OR (guinea[Title/Abstract])) OR (guinea bissau[Title/Abstract])) OR (guyana[Title/Abstract])) OR (haiti[Title/Abstract])) OR (honduras[Title/Abstract])) OR (india[Title/Abstract])) OR (indonesia[Title/Abstract])) OR (iran[Title/Abstract])) OR (iraq[Title/Abstract])) OR (jamaica[Title/Abstract])) OR (jordan[Title/Abstract])) OR (kazakhstan[Title/Abstract])) OR (kenya[Title/Abstract])) OR (kiribati[Title/Abstract])) OR (democratic people s republic of korea[Title/Abstract])) OR (kosovo[Title/Abstract])) OR (kyrgyz republic[Title/Abstract])) OR (laos[Title/Abstract])) OR (lebanon[Title/Abstract])) OR (lesotho[Title/Abstract])) OR (liberia[Title/Abstract])) OR (libya[Title/Abstract]))		

	OR (madagascar[Title/Abstract])) OR (malawi[Title/Abstract])) OR (malaysia[Title/Abstract])) OR (maldives[Title/Abstract])) OR (mali[Title/Abstract])) OR (marshall islands[Title/Abstract])) OR (mauritania[Title/Abstract])) OR (mexico[Title/Abstract])) OR (micronesia, federated states of[Title/Abstract])) OR (moldova[Title/Abstract])) OR (montenegro[Title/Abstract])) OR (morocco[Title/Abstract])) OR (mozambique[Title/Abstract])) OR (myanmar[Title/Abstract])) OR (namibia[Title/Abstract])) OR (nepal[Title/Abstract])) OR (nicaragua[Title/Abstract])) OR (niger[Title/Abstract])) OR (nigeria[Title/Abstract])) OR (federal republic of nigeria[Title/Abstract])) OR (north macedonia[Title/Abstract])) OR (pakistan[Title/Abstract])) OR (papua new guinea[Title/Abstract])) OR (paraguay[Title/Abstract])) OR (peru[Title/Abstract])) OR (philippines[Title/Abstract])) OR (russian federation[Title/Abstract])) OR (rwanda[Title/Abstract])) OR (samoa[Title/Abstract])) OR (sao tome and principe[Title/Abstract])) OR (senegal[Title/Abstract])) OR (serbia[Title/Abstract])) OR (sierra leone[Title/Abstract])) OR (solomon islands[Title/Abstract])) OR (somalia[Title/Abstract])) OR (south africa[Title/Abstract])) OR (south sudan[Title/Abstract])) OR (sri lanka[Title/Abstract])) OR (st lucia[Title/Abstract])) OR (st vincent and the grenadines[Title/Abstract])) OR (sudan[Title/Abstract])) OR (suriname[Title/Abstract])) OR (syrian arab reublic[Title/Abstract])) OR (tajikistan[Title/Abstract])) OR (tanzania[Title/Abstract])) OR (thailand[Title/Abstract])) OR (democratic republic of timor leste[Title/Abstract])) OR (togo[Title/Abstract])) OR (tonga[Title/Abstract])) OR (tunisia[Title/Abstract])) OR (turkey[Title/Abstract])) OR (turkmenistan[Title/Abstract])) OR (tuvalu[Title/Abstract])) OR (uganda[Title/Abstract])) OR (republic of uganda[Title/Abstract])) OR (ukraine[Title/Abstract])) OR (uzbekistan[Title/Abstract])) OR (vanuatu[Title/Abstract])) OR (venezuela[Title/Abstract])) OR (vietnam[Title/Abstract])) OR (west bank[Title/Abstract])) OR (yemen[Title/Abstract])) OR (zambia[Title/Abstract])) OR (republic of zambia[Title/Abstract])) OR (zimbabwe[Title/Abstract])) OR (republic of zimbabwe[Title/Abstract]) Filters: from 2007 - 2023		
5	Search #1 AND #2 AND #3 AND #4	8,391	14:43:34

Table S2. Intervention programs and their effectiveness on child mental health outcomes used in studies included in the scoping review on behavioral interventions for treatment and prevention of child mental health problems in low- and middle-income countries, 2007-2022.

**Author, year**	**Name of Intervention Program**	**Details about the intervention (use of theory, type, format of delivery, person who delivered the intervention and duration)**	**Child-specific objective of the intervention program**	**Child-related outcome and Intervention effectiveness**	
				**Significant effect**	**No significant effect**
Bemanalizadeh et al., 2022	Care for Child Development (CCD) Parenting	• Intervention intensity: five 45-minute educational group sessions offered pregnancy through 1-year post-partum.<br>• Intervention focus: parenting skills<br>• Interventionist: trained certified healthcare provider	To improve children's development and socio-emotional behavior problems	• Anxiety /depression	• Externalizing problems & internalizing problems and<br>• Syndrome scales including emotionally reactive, somatic complaints, withdrawal, sleep problems, attention problems, aggressive behavior, and other problems
Chu et al., 2022	Group executive functioning and online parent training (GEF-OPT)	• Used the Training Executive, Attention, and Motor Skills (TEAMS) framework.<br>• Intervention intensity: eight 90-minute sessions, with separate groups for children and parents, each consisting of four to six families.<br>• Parents underwent a 30-minute session on ADHD and behavior management skills, followed by helping their children with homework post the EF training class.	To reduce the levels of child core ADHD symptoms and to improve executive functioning, peer relationship, learning, and social function	• ADHD symptoms<br>• Parent- rated inattentive & ODD<br>• Teacher- rated inattentive & ODD<br>• Total score	• Hyperactive-impulsivity and the total score in parent-rated SNAP-IV scales (a mobile app, Swanson Nolan and Pelham, Version IV, used to measure ADHD)

		• Child groups, typically led by a team of three staff members (one senior psychologist and two graduate students), and parent groups supervised by three pediatricians specializing in child healthcare.			• Hyperactive-impulsivity in teacher-rated SNAP-IV scale
Erdemir E., 2022	Preschool Education Program (PEP)	• A summer preschool education program implemented during the 50-day period.<br>• Children received a combined total of 265 hours of intervention.	To promote developmental well-being and school readiness of children from forced displacement, to improve social competence skills and decrease in internalizing/externalizing behavior problems	• Emotion regulation scores<br>• Internalizing and externalizing behavior problems	-
Ndetei et al., 2022	Life Skills Training	• Children received eight hours of life-skills training distributed evenly across four weeks, utilizing a program developed by the Ministry of Education.<br>• The trainers were experienced teachers who had received additional training in life-skills education.	To improve mental health and academic performance	• Attention problem	• Internalizing problems<br>• Externalizing problems<br>• Total problems
Tahan et al., 2022	Animal-Assisted Therapy (AAT)	• Eight sessions of AAT, with each session lasting 90 minutes	To alleviate anxiety	• Total score of anxiety<br>• Subscales anxiety of separation<br>• Generalized anxiety<br>• Agoraphobia<br>• Social phobia	• Obsession

Zhu C., 2022	Musicotherapy & Cognitive Behavioral Intervention	• 16-week treatment with musicotherapy and cognitive behavioral intervention, involving 60-minute sessions five times a week.<br>• Cognitive behavioral therapy lasted for 16 weeks, while musicotherapy, conducted in groups of five, occurred once a week for 45 minutes over the same duration.	To improve cognitive ability of children with ADHD	• Attention deficit, hyperactivity-impulsiveness,<br>• ADHD- total scores	-
Altafim et al., 2021	Adults and Children Together (ACT) Raising Safe Kids Parenting program	Used social learning theory, cognitive-behavioral principles, and developmental theory.<br>• Total 8 weekly sessions, 2 hours/session<br>• Delivered by psychologists trained and certified as ACT facilitators	To support child behavioral well-being and to protect children from violence	• Internalizing and externalizing problems	-
Barik et al., 2021	Parent Child Interaction Therapy	• Therapy for a total of 5 sessions<br>• Each session lasting 1 hour conducted twice a week and consisted of a 30-minute Child Directed Session followed by a 30-minute Parent Directed Session	To reduce disruptive behavior level	• Disruptive behavior	-
Dowdall et al., 2021	Dialogic Book Sharing program	• Eight weekly sessions lasting 60 to 90 minutes each, conducted over consecutive weeks.<br>• Sessions accommodated groups of four to six caregivers, with the two facilitators having previous involvement in book-sharing intervention studies and completing basic schooling<br>• Led by trained facilitators,	To improve children's cognitive and socioemotional development	• Dysregulative strategies	• Child defiance behavior<br>• Aggression subscale<br>• Prosocial behavior
Goudarzi et al., 2021	Play Therapy	• Nine sessions of play therapy training, with two 30-minute sessions per week.<br>• Training provided on an individual basis, with parents accompanying their children.	To reduce anxiety and specific learning disorders of elementary-school students	• Anxiety	-

Daryabeigi et al., 2020	Cognitive-Behavior Therapy	• Combined techniques such as coping tasks, relaxation training, role-plays, and practices and rewards.<br>• Included 16 one-hour sessions and twice-a week.<br>• First eight sessions focus on therapeutic program, and the next 8 sessions focus on active engagement of children with skills-learned.	To teach children to recognize signs of anxiety and how to cope with anxiety-inducing situations and to reduce internalizing problems of children with externalizing disorders	• Internalizing behavior<br>• Symptoms such as withdrawal/depression, somatic complaints, anxiety/depression	-
Rivero et al., 2020	FunFRIENDS	• 14 sessions lasting 90 to 120 minutes each, which included:<br>a) 10 weekly group sessions for children.<br>b) 2 sessions involving parents/guardians, teachers, and community members.<br>c) 2 behavior reinforcement sessions, with the first occurring a month after the 10th meeting and the second three months later.<br>• All intervention activities were conducted by two trained psychologists following the program's protocol.	To reduce the symptoms of anxiety in children and evaluate the effects on the prosocial behavior	-	• Internalizing problems<br>• pro-social behaviours
Zhang et al., 2020	Community-based Family Workshop	• Intervention duration for six months.<br>• Both children and one of their primary caregivers attended the workshop together.<br>• Workshop followed a group-based integrated approach with four stages and a total of 12 sessions.<br>• Each session lasted for 2 hours and was conducted every two weeks.	To improve the social adaptation among left-behind children	Emotional symptoms<br>Peer problems<br>Total difficulties score<br>Hyperactivity-inattention in boys<br>emotional symptoms (P=0.048) in girls.	-

Maselko et al., 2020	The Thinking Healthy Program, Peerdelivered (THPP)	• More extensive psychosocial intervention<br>• Involved 18 group-based booster sessions spanning from 7 to 36 months after childbirth. Initially, the first six sessions occurred on a monthly basis, and then every 2 months until the 36-month mark.<br>• Facilitated by the same community-based peer volunteers, specifically married women residing in the same community as the women experiencing depression, who generously volunteered their time.	To improve child development	-	• Emotional symptoms<br>• Conduct problems, hyperactivity,<br>• Peer problems, and<br>• Pro-social behavior
Ward et al., 2020	Parenting for Lifelong Health for Young Children	• Facilitators were trained high school education.<br>• Sessions include positive relationships and reinforcing desirable behavior. Later sessions focus on limit-setting techniques, including instructions, household rules, routines, and nonviolent discipline methods like redirection, ignoring, timeouts, and consequences for undesirable behavior.<br>• Practice skills of 12 three-hour sessions and at-home practice with their children.	To reduce conduct problems	• Positive child behavior	-
Edrissi et al., 2019	Tuning in to Kids (TIK) group parenting program<br><br>Tuning in to Kids Parenting	• Six session followed by two booster sessions at monthly intervals.	To reducing preschool children's anxiety	• Parent-reported anxiety	-

Khademi et al., 2019	Positive Parenting Program (Triple-P)	• It is parenting and family-support strategy, and has origins social learning theory.<br>• Parents engaged in weekly 2-hour sessions over a span of 4 weeks, followed by an additional 4 phone-based sessions.<br>• Training included 12 to 15 participants per group.<br>• Training was given by experienced psychiatrist.	To reduce the symptoms of ADHD in preschoolers	• ADHD<br>• Inattention<br>• Hyperactive<br>• Opposition al behavior<br>• Depression<br>• Stress	-
Morshed et al., 2019	Individual and Group Play Therapy	• Individual sessions lasted 45 minutes, and group sessions were 60 minutes each.<br>• Play therapy occurred over eight weekly sessions.	To reduce symptoms of oppositional defiant disorder	• Parent report ODD symptoms<br>• Teacher report ODD symptoms<br>• Result is for both individual and group approaches	-
Pirnia et al., 2019	Parent-Child Interaction Therapy (PCIT)	• Conducted in two phases;<br>– Child-directed interaction phase: Enhancing communication skills<br>– Parent-directed interaction phase: Practicing interactive discipline.<br>• Duration: Conducted over 12 sessions.	To reduce aggression and cortisol level in children	• Aggression	-
Akcan et al., 2018	Aggressive Behavior Prevention Program (ABPP)	• Conducted 25 in-class activities with children.<br>• Duration: Over a 12-week period.	To prevent aggressive behavior in kindergarten children	• The Aggressiveness subscale of the Eyberg Child Behavior Inventory (ECBI),<br>• The subscales of overt and relational	• Peer victimization

				aggression in the Preschool Social Behavior Scale-Teacher Form	
Derakhshanpour et al., 2017	Psychosocial Interventions	• Parenting skills instruction provided through 6 sessions.<br>• Two sessions dedicated to anger management and discussions on child abuse and the negative effects of physical discipline.	To reduce the frequency of child abuse and decrease the problems of children	• Mental Health (GHQ score)<br>• Conduct problem<br>• Hyperactivity<br>• Peer problems	• Emotional symptoms<br>• Socialization
Goncalves etal., 2017	Massage and Storytelling	Massage Group intervention:<br>• Teacher training: 4 hours before weekly 50-minute classes.<br>• Children's activities: Practiced massaging each other, starting with 10 minutes daily after the first class.<br>Storytelling Group intervention:<br>• Teacher training: 4 hours.<br>• Children's activities: Chose books, listened to stories, and shared thoughts in weekly 50-minute classes. Additionally, paired up daily for 10 minutes to retell the story.	To reduce aggressive behaviors at school and academic performance of elementary school children	• Aggressive behaviors	-
Huang et al., 2017	Evidence-based early childhood interventions using Professional Development	ParentCorps Professional Development included:<br>**FUNdamentals Training:**<br>• Duration: 5 days.<br>• Objectives: Build knowledge of evidence-based practices (EBPs), motivate change, boost confidence, and foster a sense of community among teachers.<br>• Activities: Reflection on assumptions, alignment with current practices and goals, learning EBPs matching values.	To improve child social competence	• Social competence<br>• Emotion regulation<br>• Prosocial skills<br>• Problem behaviors	• Externalizing problems<br>• Parent-rated internalizing problems

		• Support: US$50 stipend for travel and time expenses (outside school hours).<br>**Coaching Sessions:**<br>• Frequency: Thirteen weekly group coaching sessions (1-1.5 hours each).<br>• Facilitators: Conducted by pairs of mental health professionals.<br>• Focus: Helping teachers effectively implement EBPs in classrooms.<br>• Approach: Group coaching during breaks or after school, considering cost-effectiveness and aligning with Ugandan collective culture.			
Leung et al., 2016	Happy Parenting Program	• Duration: Eight weekly 2-hour group sessions.<br>• Activities: Mini lectures, group discussions, role-playing, and homework assignments.<br>• Content: Covers improving parent-child relationships, teaching new skills, and managing child behavior.<br>• Facilitators: Led by two educational psychologists, assisted by two trainee educational psychologists.	To reduce child behavior problems	• Disruptive behaviors problem<br>• Disruptive behaviors intensity<br>• Both measured using Eyberg Child Behavior Inventory	-
Li et al., 2016	Social Skills Training & Facilitated Play Program	• Sessions: Twice weekly for one hour each over a 7-week period.<br>• Facilitator: Female group leader trained by two senior authors with a background in early childhood education.	To promote social interaction, prosocial behaviours and sociocommunicative skills	• Peer interactions<br>• Prosocial behaviors<br>• Social-communicative competence	-
Deeba et al., 2015	Enhanced Huggy Puppy	• Children in both groups received a small stuffed animal named Aduri. Children instructed to keep the toy at all times, except during baths and school.	To reduce symptoms of PTSD, internalizing problems, and improve cognition	• Anxiety	• PTSD (but significant in the enhanced HPI

		• Care Routine: Twice daily—before school and bedtime.<br>• Enhanced HPI Group: Introduced to positive statements for self, others, and the future.<br>• Encouraged to use or create their own positive statements. Caregivers attended as observers, understanding tasks and reminding children.<br>• Standard HPI Group: Caregivers reminded children to care for the toy only.<br>• Caregiver in Enhanced group responsible for both toy care and encouraging children to use positive statements. Leaflet with statement examples provided to caregivers.			group from Time 1 to Time 2)<br>• Depression
Maselko et al., 2015	Thinking Healthy Program	• Intervention administered by community health workers during 16 home visits.<br>• Training for these workers began in the last month of pregnancy and extended for 12 months postpartum.<br>• The intervention, based on a psychosocial model, aimed to foster positive and healthy thinking for both mothers and babies.	To reduce perinatal depression and its negative impact on child development	**SCAS anxiety components such as:**<br>• Panic and Agoraphobia<br>• Obsessive-compulsive	• Total difficulties measured using the Strengths and Difficulties Questionnaire (SDQ)<br>• Anxiety as measured by the Spence Children's Anxiety Scale (SCAS)<br>**Strength and Difficulty Questionnaire components such as:**<br>• Emotional,

					• Conduct problem,<br>• Hyperactivity,<br>• Peer problems,<br>• Prosocial<br><br>**SCAS anxiety components such as:**<br>• Separation<br>• Injury fear<br>• Social phobia<br>• General anxiety
Puffer et al., 2015	Parents Make the Difference	• A 10-session intervention<br>• Lay librarian facilitators led weekly 2-hour sessions, working in pairs and engaging groups of 20-35 caregivers.	To improve children's cognitive, emotional, and behavioral wellbeing	-	Child wellbeing outcomes measured using SDQ caregiver report such as:<br>• Conduct problem<br>• Emotional<br>• Hyperactivity
Eloff et al., 2014	Intervention to Promote Resilience in young children	• Consisted of 24 weekly group sessions facilitated by community care workers.<br>• It initiated with 14 sessions involving separate participation of mothers and children, followed by 10 interactive sessions.	To promote resilience in young children living with their HIV-positive mothers	• Externalizing behaviors- parent reported<br>• Anxiety-child reported	• Internalizing behaviors<br>• Socialization<br>• Both as reported by parents<br>• Depression- child-reported
Leung et al., 2013	The Triple P Level 4 Group Version	• The program consisted of eight sessions, incorporating 2-hour group meetings and two follow-up phone contacts.<br>• Parents engaged in group sessions that included mini-lectures, discussions, role-play, and exercises.	To reduce child behaviour problems	• Child behavior problems Intensity<br>• Child behavior problems problem<br>• Both measured using the Eyberg	-

		• Facilitated by female social workers accredited as Triple P Level 4 group providers.		Child Behavior Inventory	
Baker-Henningham et al., 2012	Incredible Years Teacher Training Program	• The staff from the intervention school took part in a total of eight full-day workshops.<br>• Among these, four workshops were part of their routine in-service training days, with teachers divided into two groups of 25-30 participants. The remaining four workshops were conducted in smaller groups, each consisting of 12-16 teachers.<br>• The intervention involved the presentation of video vignettes over eight days, along with additional role-plays, practical activities, and small group exercises.	To reduce child conduct problems and promote social skills	• Conduct problems<br>• Hyperactivity<br>• Peer problems	• Emotional problems<br>• Prosocial
Walker et al., 2010	Psychosocial stimulation	• The intervention for LBW-T infants and mothers involved weekly home visits for the first 8 weeks and continued up to 24 months.<br>• Community health workers received 2 weeks of child development and intervention training before each phase.<br>• Initial 1-hour visits in the first 8 weeks focused on enhancing maternal responsiveness, while 30-minute visits from ages 7 to 24 months introduced play techniques and encouraged positive reinforcement.<br>• Mothers were urged to include play in daily routines.	To improve cognition and behaviour of children	• Total difficulties measured using SDQ	-

Ozer et al., 2009	Oportunidades Conditional Cash Transfer Program	• Program benefits were extended to eligible households starting from April 1998 to November 1999, contingent upon meeting Oportunidades program requirements.<br>• Families enrolled in Oportunidades received cash transfers bi-monthly, resulting in a 25% rise in their overall household income.	To reduce the negative effects of poverty on children's development and improve children's behavior	• Aggressive /oppositional symptoms	• Anxiety /depressive symptoms or total problem behaviors<br>• Total symptoms<br>• All measured using Behavior Problems Index
